# Attributions and private theories of mental illness among young adults seeking psychiatric treatment in Nairobi: an interpretive phenomenological analysis

**DOI:** 10.1186/s13034-018-0229-0

**Published:** 2018-06-01

**Authors:** Judy Wanjiru Mbuthia, Manasi Kumar, Fredrik Falkenström, Mary Wangari Kuria, Caleb Joseph Othieno

**Affiliations:** 1 Department of Psychiatry, College of Health Sciences, University of Nairobi, P.O.Box 19676, Nairobi, 00202 Kenya; 2 Research Department of Clinical Health and Educational Psychology, University College London, Gower Street, London, WC1E 6BT UK; 3 Department of Behavioural Sciences and Learning, Linköping University, Linköping, Sweden

**Keywords:** Youth in psychiatric facilities, Attributions of mental illness, Locus of control, Private theories interview, Stigma

## Abstract

**Background:**

Mental illness affects every segment of population including young adults. The beliefs held by young patients regarding the causes of mental illness impact their treatment-seeking behaviour. It is pertinent to know the commonly held attributions around mental illness so as to effectively provide psychological care, especially in a resource constrained context such as Kenya. This helps in targeting services around issues such as stigma and extending youth-friendly services.

**Methods:**

Guided by the private theories interview (PTI-P) and attributional framework, individual semi-structured interviews were carried out with ten young adults of ages 18–25 years about their mental health condition for which they were undergoing treatment. Each interview took 30–45 min. We mapped four attributions (locus of control, stability, controllability and stigma) on PTI-P questions. Data was transcribed verbatim to produce transcripts coded using interpretive phenomenological analysis. These codes were then broken down into categories that could be used to understand various attributions.

**Results:**

We found PTI-P to be a useful tool and it elicited three key themes: (a) psychosocial triggers of distress (with themes of negative thoughts, emotions around mental health stigma and negative childhood experiences, parents’ separation or divorce, death of a loved one etc.), (b) biological conditions and psychopathologies limiting intervention, and (c) preferences and views on treatment. Mapping these themes on our attributional framework, PTI-P themes presented as causal attributions explaining stigma, locus of control dimensions and stability. External factors were mainly ascribed to be the cause of unstable and uncontrollable attributions including persistent negative emotions and thoughts further exacerbating psychological distress. Nine out of the ten participants expressed the need for more intense and supportive therapy.

**Conclusion:**

Our study has provided some experiential evidence in understanding how stigma, internal vs external locus of control, stability vs instability attributions play a role in shaping attitudes young people have towards their mental health. Our study points to psychosocial challenges such as stigma, poverty and lack of social support that continue to undermine mental well-being of Kenyan youth. These factors need to be considered when addressing mental health needs of young people in Kenya.

## Background

Mental illness affects every segment of the population. Mental health issues among the youth can negatively impact the national development of a country. More so, the beliefs held by the community about the causes of mental illness are likely to impact individual treatment-seeking behavior [1–4]. Available literature from developing countries show that acceptance of help with mental health issues and engagement with services can be affected by various factors such as belief in evil spirits and stigmatization of mental health problems [2, 4–7]. Young people’s attitudes towards peers with mental illnesses has been studied and the findings suggest that young people differentiate between perceptions of how dangerous and fear provoking the individuals might be [8]. However, no research has explored young peoples’ beliefs and attributions associated with their illnesses. Cultural components such as social attitudes, peer group rules, religious beliefs, family morals, and other socio-cultural factors strongly contribute to the behavior and attributions towards mental illness held by the youth [9]. Data on years lived with disability (YLD) demonstrate that, in Kenya, the burden of mental health is significant and access to specialist care is limited [10, 11]. The gaps in meeting mental health needs and providing services for young adults are worrying, given increasingly high levels of depression and incidents of suicide by young adults in Kenya [12–14].

Young adults’ attributions of mental illness are not well-researched; hence, we know very little about their patterns of help-seeking behavior or their commonly used description of distress. Our study is an exploratory step towards understanding young adults’ expression of distress, their attributions associated with mental illness, and factors that prevent or contribute to management of mental disorders. By gathering mental illness-related attributions held by the young adults, we made it possible to extrapolate aspects of their psychotherapy care which need further strengthening. Exploring the beliefs young people hold about their illness and pathways to cure are important steps to facilitating early access to mental health services and improving psychological wellbeing. This includes alerting the practitioners about possible barriers that hinder positive psychotherapy outcome. Through this research we gained a critical knowledge piece that will provide insight into barriers young adults encounter in seeking mental health services. To achieve this, we used two theoretical lenses to review subjective appraisal from young adults: attributional framework and the private theories interview.

### The private theories interview patient version (PTI-P)

The private theories interview [15] is an interview that was developed in the context of psychotherapy research, and is used to study psychotherapy patients’ subjective beliefs about their problems, their causes, and any ideas they may have about what would be needed for them to feel better. We used the patient version of the interview (PTI-P). We superimposed this interview on an attribution model developed by Weiner [16]. We used these two frames to capture the interviewee’s attempts to give meaning to their interpersonal, psychic, and somatic distress, while including these experiences in the patient’s private context of meaning. The PTI-P is a semi-structured, brief qualitative interview, which can be used to understand participants’ personal assumptions of treatment, mental health or illness qualitatively. We adopted the PTI-P, developed by [17], as it is with minimal changes. The attribution-focused questions were superimposed on the PTI-P, by adding extra probes to questions in the PTI-P. See Table 2 for the newly designed attribution-focused questions.

### The attributional framework

Depression, anxiety, and stress are commonly associated with negative thinking and attributions. We outlined our attribution framework from the original attributional framework done by Bernard Weiner [18]. Attribution refers to the assessments of the cause of an action or behavior [19]. It also refers to the internal (thinking) and external (talking) process of interpreting and understanding what is behind our own and others’ behaviors. Attribution theory explains an occurrence and determines the cause of the happening or behavior. It starts with the idea that individuals are driven to understand the causes of the happenings or behavior and that this desire allegedly grows out of individuals’ wish to understand, foresee, and control the environment [20–22]. The attributions are known to be of different types. According to Weiner et al. [23], there are three dimensions of causal attributions which include the following.

#### Locus of control (internal vs. external)

A person’s belief that the events which occur in life are either a result of personal control and efforts or an outside force like luck or fate is referred to as locus of control (LoC). *Controllable* vs. *uncontrollable attribution:* Weiner’s controllability dimension concerns a situation that is regarded as controllable if the individual is personally able to guide, influence, or prevent it. It is the extent to which the individual has control over the cause, as perceived by observers. Försterling [24] used “drunkenness” as an example to describe the controllability of causes, suggesting that “drunkenness” is perceived as a controllable cause. Causes that can neither be influenced nor guided such as a physical handicap, for example blindness, are regarded as being uncontrollable [24]. The external locus of control are often thought to be relatively uncontrollable and associated with perceived social stress that young adults might encounter [25].

#### Stability of causal attribution (stable vs. instability)

Stability is the time-based nature of causes [16]. Some causes remain stable over time while others increase or decrease. Causal attributions, when viewed as stable and unchanging as opposed to unstable and fluctuating, are directly related to a person’s expectancy of successful results [24] implying that the stability attribution makes the person less inclined to believe that his/her problems will improve. As the mental illness deteriorates in an individual, it perpetuates an irrational outcome and damages self-governing functions. This could be explained by the knowledge that mental-behavioral or internal stigmas are normally considered unstable or reversible, while physically based stigmas are perceived as stable, or irreversible [26]. Stability and instability of attributions refer to how fixed or how flexible the mental schemas associated with these can be. Unstable attributions may help with the motivation to work in treatment, whereas stable attributions may lead to hopelessness and not seeking treatment. The more stable an attribution is, the harder it is to change it [16]. Inferences on the stability and instability of an attribution depends on how controllable or uncontrollable one experiences an event or an individual attribute to be; it is also contingent on whether one views the event or attribute it from an internal or external locus of control.

## Attribution of stigma (internalized vs. externalized)

Stigma is defined as a social scratch that leads to questioning of associates of a group, such as people with mental illness [27]. According to Rüsch et al. [28], the negative properties of stigma among individuals with mental illness lessen self-esteem and health care seeking behavior, and increase discrimination. The tendency towards self-stigma has been documented in Sub-Saharan African patients and along this are also religious and supernatural attributions given to mental health conditions in the form of punishments [29]. Experiences of stigma catalysed by self-stigma revolve around experiences of devaluation, exclusion, and disadvantage [30]. Moreso, mental illness stigma is one factor that hinders seeking care by distressed people, hence undermining the service system [31]. The individuals are not only troubled by the external mental illness stigma but also by the self-stigma, leading to low self-esteem and self-efficacy [32].

## Methods

### Setting

The study took place at the Kenyatta National Hospital’s Youth clinic. The clinic runs every weekday from 7.30 am to 5.00 pm, and roughly ten new patients are registered each week and around fifteen more new patients are added during school holiday periods. It is overseen by resident psychiatrists, psychiatric nurses, and psychotherapists including clinical psychology interns. The services are free to all youth regardless of type of diagnosis, and the clinic also provides outreach services for HIV testing and counselling (HTS). This setting was chosen because it serves nationwide referrals and walk-in patients who are predominantly fluent in either English or Kiswahili. Most clients are referred from various schools, colleges, and universities, and it is also a clinic for walk-in cases and emergencies.

### Participants

Our participants were between ages 18 and 25 years. 18 years is the legal age for an adult in Kenya [12], while 25 is the age limit for patients attending the youth clinic (Table 1).

**Table 1:** The Socio-demographic information

Participant no.	Age	Gender	Level of education	Diagnosis
Patient 01	20	Male	University	Major depressive disorder
Patient 02	19	Male	High school	Substance induced psychosis
Patient 03	18	Female	College	Secondary enuresis
Patient 04	22	Female	University	Schizoaffective disorder
Patient 05	19	Female	High school	Depression
Patient 06	24	Female	University	Depression and epilepsy
Patient 07	20	Female	High school	Conduct disorder
Patient 08	22	Male	College	Somatic disorder
Patient 09	19	Male	High school	Substance induced psychosis
Patient 10	20	Female	College	Post-traumatic stress disorder

### Sampling

In this exploratory study, we purposely selected young adults who were seeking psychiatric treatment at the clinic, were willing to give consent, and had fluency in Kiswahili or English (the two official languages spoken in Kenya). Assessments from the psychiatrists and clinical psychologists were used to determine the severity of the patients’ mental illness. Two patients who were eligible for the study were excluded because both had severe psychosis in addition to limited fluency in English and Kiswahili. None of the participants we approached for the study declined to give consent and participate.

### Ethical approval and considerations

We received approval from the Kenyatta National Hospital and University of Nairobi Ethical Review Committee (no. KNH/UoN P105/02/2015). The participants were informed verbally and also provided with a written document about the purpose of study. In addition, consent to audio-record the interview was sought. Participants were informed that participation or refusal to take part in the study would not affect their current contact with the clinic. No rewards were given for participation.

### Instruments

A brief socio-demographic questionnaire was used to capture key demographic information. These included the age of the participant, Kiswahili and English literacy levels, educational level, and gender of the participants. These were gathered to synthesize the information with their interviews. Data was gathered by use of semi-structured interviews with open-ended questions encouraging exploration of experiences within the conceptual framework used. The five questions from the *Private Theories Interview*-*Patient version* (PTI-P) were the primary interview questions. We developed an *Attributions Focused Question guide* that was embedded within the PTI questions as probes in such a way as to elicit attributions. The probes were designed to get the participants to elaborate on the ‘how’, ‘why’, and ‘when’ associated with the five private theories questions. The attribution-focused question guide was mapped onto these questions to create a subset of categories that explain the attributions used by participants in understanding their problems and thinking about how these could be resolved. Given that mental illness continues to be a highly stigmatizing condition in Kenya [33], we included stigma as a category of attributions to see how our participants navigate it.

Since the PTI-P was first developed in Sweden, we were concerned how well these questions would fit the needs of our Kenyan young participants. In order to enhance cultural sensitivity and adapt the questions to the Kenyan context, the question-guide was translated into Kiswahili. The first author, who is fluent in Kiswahili and English, translated with the help of an English-Kiswahili dictionary to ensure that the meaning of each word was retained. Two Kenyan trainee-psychologists also gave suggestions on a few semantic adjustments. The adjustments were only linguistic in nature. Given that these were semi-structured questionnaires there was no need to formally validate the tools. Instead, we tested their cultural sensitivity in pilot interviews. We used interpretative phenomenological analysis (IPA) [34]—a qualitative technique to analyze our data. IPA was chosen because our goal was to magnify the subjective experiences, both tone and tenor expressed by our participants while interpreting their idioms of distress, attributional patterns, and highlighting their barriers to mental health care.

These questions resulted in the attribution framework as shown in Table 2 below and embody each locus, stability, and stigma probes mapped on PTI-P. The questions were the following:

**Table 2:** Private theories interview patients’ version and attributions focused questionnaire guide

Private theories questions	Attributions focussed domains
What is it that leads you to seek treatment today?	Internal locus of control External locus of control
What are your thoughts about the psychological issues you are experiencing?	Controllability Uncontrollability
Tell me about some or other important experiences or events in your life that you associate with your difficulties and how these problems	Stability Instability
In relation to the problem (MI) how do you see yourself and others around you?	Experiencing stigma Not experiencing stigma
What do you desire that would ease your pain/distress?	Desired treatment plan or cure

1. What is the problem?
2. How did the problems arise?
3. How can the problems be remedied?
4. What has changed?
5. What is your view of others and yourself?

Each of these questions was paired with a domain from the attribution theory. AFQ provided a deepening and expanding of the PTI-P framework and the scope of the analyses. The domains adapted from Wieners attribution theory were locus of control (internal vs. external), controllability (controllable vs. uncontrollable) stability (stable or unstable), and stigma as an independent domain.

### Data collection

Three pilot interviews were conducted at the youth clinic to test the conceptual framework and gauge participants’ reaction to the interview questions. The first author was trained in qualitative interview techniques by her senior mentor MK and had regular supervision with all her mentors on using IPA as well as analyzing the data thematically. The participants in distress were encouraged to continue with psychotherapy, and a referral mechanism was built in the study if anyone had self-harming thoughts or was at a risk of harming themselves or others. Once these procedures were identified, individual interviews took place in the counselling room at the youth clinic before or after the participants’ counselling session. English or Kiswahili language was used as per the participant’s preference. The first author conducted the audio-recorded interviews with one interviewee at a time. The interview duration ranged from thirty to 40 min. Data collected was safely stored without any identifiers to ensure confidentiality of the participants.

### Data analytic plan

The recorded material was transcribed from the audio recorder to a MS Word document. The first step was to ensure that all experiential material about PTI-P and Attribution Focused questions were adequately answered in the data. The second step was to break the data as per the IPA framework. This was done because of the following reasons: (i). IPA is consistent with research aims since it is committed to the *examination of how people make sense of their major life experiences* [35], (ii). It is a phenomenological approach *focusing on exploration of experiences in its own terms* instead of attempting to reduce it to predefined or overly abstract categories. This means *that it is interpretative in that the researcher tries to make sense of the participants’ experiences*, and (iii). IPA is concerned with personal experiences and involves interpretation, with ample consideration of a given context.

#### IPA as a scientific principle

IPA is idiographic in nature. It is concerned with revealing something about the experience of each of the individuals involved and is able to give a detailed conclusion about the participant group. Our third and next step was to ensure that we used IPA as a method in the service of teasing out PTI information and how young adults make attributions regarding their own well-being. The approach is committed to detail in-depth analysis as well as to understand how a particular experiential event or relationship (phenomenon) has been understood from a particular context by different individuals or groups.

#### IPA as a specific method in this study

We used IPA in the following ways: by carrying out verbatim transcription of the semantic content of each interview based on audio recording and followed by reading and re-reading of the content, while searching for richer, detailed sections and for contradictions and inconsistencies. At the initial noting stage, the first author and her supervisors identified specific ways the participants spoke of an issue, described what mattered to the participants, and the meaning of these things. This identified each participant’s emergent themes and the connections/interrelations of the themes for each of the ten participants. Mapping of themes was done to connect and fit the themes in relation to the research questions. With each step, every individual participant’s core themes were tallied with other participants’ and we ensured that the analysis maintained a strong interpretive focus. The core themes were later merged with the attribution dimension and the PTI-P framework to make sense of the bigger picture. At this point, our key question was which themes were being articulated by our participants and where did these fit-in vis-à-vis identified attributions. We present themes emanating out of IPA from the private theories interview in the results section and in our discussion section we reflect on how these themes map onto attributions framework as a whole.

## Results

Our results are presented in a twofold process. We highlight the themes that were drawn from the PTI-P: psycho-social triggers, biological origins, and preference for combined treatment as a way of addressing stigma. Table 3 lists the core themes arranged from the most prominent to the least, as derived from frequency count among the 10 participants, while Table 2 indicates the connections between the attributions and core themes. These themes are reviewed here, starting with the psycho-social attributions.

**Table 3:** Building the connections (attributions vs. core themes) (to be placed in page 30, before internal and external locus of control)

Interview questions	attribution dimensions	Core themes
*What is it that leads you to seek treatment today?*	Internal locus of control or dispositional attributions: based on behaviour within the client	Negative emotions and thoughts misconduct behaviour Transitional challenges-from teen to adult life Poor performance in school Self-stigma and shame of disclosure
	External locus of control or situational attributions: Based on behaviour (from others) to the individual	Negative childhood Experiences Strained relationships with parents and other family members Rejection from others and stigma Lack of finances Decline in social life
*What are your thoughts about the psychological issues you are experiencing?*	Controllability: if the individual is personally able to guide, influence or prevent the situation	Negative emotions and Thoughts
	Un-controllability: if the individual is personally not able to guide, influence or prevent the situation	Negative childhood experiences Strained relationships with parents and other family members Rejection from others and stigma Lack of finances Decline in social life
*Tell me about some (other) important experiences or events in your life that you associate with your difficulties and how the problems began.*	Stability: unchanging causes	Death of loved ones
	Un-stable: changing/fluctuating causes	Negative emotions and thoughts
*In relation to the psychological issues, what is your view on others and yourself?*	Stigma from others and self	Self-stigma
		Stigma from others
*What do you think is needed for your illness to be cured or might ease your pain?*	Treatment preference	Need for therapy
		Need for medication

### Psychosocial triggers of distress

Our participants were concerned about various psychosocial triggers that adversely impacted their lives. Employing IPA, we identified a number of thoughts and experiences as being the prominent causes of our participants’ worries and distress.

#### Negative thoughts and emotions

The participants shared in their interviews that negative thoughts and emotions were the core reasons for their illness and distress. Adverse experiences created a spiral of negative thoughts and emotions about themselves and the world around them. The PTI question 1 was most reflective of this spiral thinking that our participants struggled to get out.

*“I had a disagreement with mum. She wants me to be like her and I cannot. She separated with my dad and now she wants me to go live with my uncle who is very tough. She is also planning to go for further studies abroad.”* 20-year old young man.

“[…] *I had a tough childhood; my brother uses drugs and abuses me. I also lost my dad at a young age…”* 22-year old female participant.

These vignettes point to grim interpersonal context that generate self-doubt and apathy in the participants.

Parental separation, unexpected death of a loved one, and protracted bereavement thereafter worsened the situation and the participants’ mental health, as experienced by the young adults interviewed. Female participants echoed such experiences more than their male counterparts. Vignettes such as the following are testament to these early deprivations and adversities which they highlighted:

*“My dad does not care. Since the illness started from childhood, he has never sent money for medication. He went and got another wife. He only sends money for food for me and my sister. But for my medication, he has never sent … money. My mother who lives with me does not work. She is a house wife and depends on the small amount send my father…”* 24-year old female participant diagnosed with epilepsy.

“[…] *I used to love my father but when my sister was born, it’s like he forgot about me. He only cared about her. I started talking to boys and eventually lost my virginity. I still feel bad about it…”* 19-year old female participant.

*“My mum died. I still don’t know how to deal with that. She was the most important person in my life. Always cheering me… I was her only child. I have no dad. I felt lost and never gotten over this. I do not understand myself anymore.”* 25-year old female participant.

At a fairly early age, the participants had to deal with situations that left them emotionally scarred. Seven of them had an early childhood experience that they attributed to be the cause of their mental illness that brought them to the hospital in the first place.

#### Adjustment and behavioral problems in school and college

This theme captured the participants’ thoughts about the need to be accepted by peers, family, and teachers. It also demonstrated the difficulty one may have in finding a friend who would guide and influence in a positive way. As we learnt in our interviews that the participants were mostly connected with difficult conduct-related behaviors (externalizing tendencies) for which the youth were seeking support. These vignettes underscore these problems:

*“My friend and I had a phone in school. During prep time, the teacher on duty caught us playing games. We have been suspended for 2* *weeks and told to go back to our parents…”* 19-year old female participant.

*“I started taking alcohol after high school. I thought it was normal for those in university to take alcohol since now you are a grown*-*up and other people especially my friends were taking it. So I thought, why not join them? I hope to stop completely as it is the cause of Bell’s palsy that I have now…”* 21-year old male participant.

#### Familial challenges and lack of support in transitioning process

Most of our male participants expressed difficulties in overcoming life transitions and alluded to absence of support in navigating resultant challenges. Six participants described the challenges of transiting from one phase of life to another i.e. from childhood to demands and expectations of youth, while some struggled with fitting in their social milieu due to mental illness. The following vignette explained these challenges further:

*“I repeated form IV then joined university where I am studying mass communication. In the first semester, I started having weird feelings and thoughts. I felt like I do not fit into the school culture. People were just having fun. Right left and center. Then I got myself in this group of girls who had money from their boyfriends and older men. I wish I did not join them. Somehow I lost my virginity…..”* 22-year old female participant.

*“I am not comfortable with my life. I have not achieved the things I have wanted to achieve. Just the way my life is going…..my career…Everything is moving slowly. Am in a stage where I want to do new things and find my own place in life”* 24-year old male participant.

*“Since I went to boarding school in class six my performance dropped and was always punished for it….”* 20-year old male participant.

As a result of challenges in transitioning to new environments (e.g. day schooling to boarding), death of a loved one, and lack of finances or strained relationships with significant others, four of them described their poor academic performance as a cause of their psychological distress. Some maintained this to be the main cause of their mental illness while others thought if they had better upbringing or did not have to face difficulties in their childhood, they would have performed much better academically.

“I used to think a lot after failing my KCSE^1^. I was wondering what next? This is when I started having too much headache and a lot of fear.” 22-year old female participant.

#### Strained relationships with parents and other family members

Four participants attributed a conflictual relationship with their caregiver as a leading cause of their psychological distress. Coming from unsupportive families, abusive parents or siblings, parent child discrimination or preferential treatment, parental divorce or marital conflicts were shared as being the primary trigger of their current psychological distress. A client reported to have hated the day her younger sister was born:

*“Dad started neglecting me and it is like all the love I had for him ended. He still prefers my sister and I feel like she is more special than me. Maybe it is because she is named after mum to my dad.”* 19-year female participant.

A client reported to have had no connection with his mum due to lack of motherly affection and attention since he was very young:

*“I grew up with my extended family since mum had travelled out of the country for further studies. When she came back, she was a stranger to me. We still do not have a relationship.”* 19-year old male participant.

The PTI question “Tell me about some (other) important experiences or events in your life that you associate with your difficulties and how the problems began” was the most relevant to this theme:

*“I stay with my mum and brother. We are not close to each other and I am not free to talk to them since they do not care about my opinion. I just keep quiet.”* 20-year old male participant.

*“I am angry at my dad. Really very angry. He listens to his relatives more than he listens to us. Like now I wanted to go further my education in UK but a sister to my dad said I should not go because I am epileptic. My dad agreed with her. He does not like supporting me. But one day I will prove them wrong. I will work hard and show them that epileptic people can do great in life.”* 24-year old female participant.

#### Stigma and rejection from significant others and a tendency towards self-stigma

Five out of ten participants attributed discrimination and stigma emanating from people around them as further triggering their mental illness and distress. Rejection, being teased, and feeling judged by relatives was common among the five participants. Peer pressure was mostly described by the participants with substance abuse.

The PTI question “In relation to the psychological issues, what is your view on others and yourself?” is illustrated here:

*“When am alone, I feel great. But when am with my mother [sic] I feel bad because my mum thinks am unimportant.”* 19 years old male participant.

*“My friends used to undermine me because my mum was old, deaf and dumb. And we were very poor. I had no friends when growing up. They hated me.”* 25-year old female participant

The inability of a parent to care, address the participants’ needs, or social problems negatively impacted the psychological wellbeing of our participants. Four out of the 10 participants interviewed shared their suffering from low self-esteem because their families did not support them or had socioeconomic or psychological problems themselves. They feared disclosing their illness or others knowing that they were seeking psychiatric help as it would bring stigma. They attributed their distress to rejection or discrimination.

*“I used to be an active child but am now introverted. I do not want my friends to know that I came for counselling. I also did not tell my mum…….. Also, when I feel like everyone knows am not a virgin. I don’t want to hang out with boys so that they do not find out about this.”* 19-year old female participant.

*“After being caught with bhang, people viewed me as a peddler making me feel so bad and couldn’t face people after that incident. My self*-*esteem was affected. Some friends deserted me.”* 19-year old male participant.

### Biological conditions and psychopathologies limiting intervention

Three of our participants shared their struggle with organic conditions such as Epilepsy, Bell’s palsy, and Psychosis (under remission).

*An Illustration from a participant with Epilepsy:*

In response to PTI question “What are your thoughts about the psychological issues you are experiencing?” this is what a participant had to say:

*“I was diagnosed with epilepsy when I was a young child. Growing up as an epileptic person is very challenging. People do not want to be associated with you, my father does not care about me. Maybe he thinks I am a burden, since he doesn’t buy my medicine. Were it not for epilepsy, I would be so happy. I have never been happy in my entire life. But I will prove people wrong. I want to show them that I can achieve my goals despite being epileptic.”* 24-year old female.

This led to experiences of anger and emotional discontent in our participant. She went on to describe her pain as being “too much to bear.” She thought that her unhappiness was due to the fact that she has always been epileptic and having to face stigma from close relatives and friends.

*An Illustration from a participant with Bell’s palsy:*

In response to PTI question “What are your thoughts about the psychological issues you are experiencing?”

*“I cannot feel one side of my mouth. It is not there. I have gone for physiotherapy but still… so my dad being a psychiatrist thought I counselling would help solve the issue. But am fine. It is only this side of the mouth that is bringing me down and I am not myself.”* 22-year old male

*An Illustration from a participant diagnosed with Psychosis:*

In response to PTI question “What are your thoughts about the psychological issues you are experiencing?”

*“..…..Then I started getting headaches. Too many fears and thoughts. When I went to hospital, the doctor said I had psychosis. Yes I have tried to Google what that means. It is not easy to live with that and when you tell people they say you are ‘chizzy’ (means ‘mad’ in Kiswahili).”* 22-year old female

From the quotes above, it is evident that in the mind of these patients there was a fear about their long term well-being and a feeling of stigmatization from other relatives that led the participants to be withdrawn.

### Preferences and views on cure

Nine out of the ten participants interviewed reaffirmed the tremendous value of psychotherapy as the most effective mode of intervention. One of our participants had had psychotherapy earlier; this prompted him to initiate therapy when the need arose. The following treatment-related preferences stood out:

#### Affirmation of psychotherapy as the most appropriate and helpful intervention

Our participants wanted concrete ways to move on from their current situation by guidance and support from a professional. It shows how several participants wanted to engage in counselling and believed that they could learn and improve their life situations with the skills they would learn during treatment.

In response to PTI interview question, “What do you think is needed for your illness to be cured or might ease your pain?”

*“My dad often takes us for counselling just to make sure all is well. Prayers are good but I prefer something tangible such as counselling.”* 22-year old male participant.

Others wanted to learn coping mechanisms—learning how to manage their feelings in a constructive way or focus on important things in their life.

*“I believe I need to control myself with regards to my anger. The only person I cannot control is my dad. So I let him be. But I need to know how to stop over reacting when I get angry.”* 24-year old female participant.

Seven participants believed that their negative childhood experiences caused their problems and continued to affect them, and these needed to be managed in order to move on with life. In this regard, a 20-year old female participant, who lost her mum at a young age made the following remarks:

*“I still do not know how to deal with her demise. I want to understand myself better and be more productive in life. I am growing old. I need to know how to deal with mum not being around.”*

She believed that working through her past experiences would lead to a more productive life and consequently enable her to be psychologically healthy. The participant stated that she needed the support from a professional in order to come to terms with her mum’s death. These participants thought that positive coping mechanisms coming from interaction with a professional psychotherapist were important in reshaping their lives. Another 19-year old male participant who had been suspended from school said that peer pressure was a cause to his psychological and emotional pain:

*“If I had listened to my inner voice that was telling me to avoid those guys, I would be so ok. I would be in school like other students. I will be attentive to my thoughts when asked to do something next time.”*

A 24-year old young woman participant considered going back to school so that she could be happy:

“*If I get the scholarship to UK, I will be happy. I want to be a better person and be busy. Being busy has helped me a lot. Now I do not concentrate on dad not buying medicine. I also do some volunteering work and get paid. Being busy helps a lot. But when idle, I get to think a lot and get angry over small issues.”*

Being involved in activities that the participants enjoyed doing and being in tune with their own feelings and thoughts were related to having a positive mental health. In this regard, the treatment offered life skills and problem-solving strategies. Professional help was emphasized over other alternative means of coping by our young participants. Involvement in activities that did not yield positive impact brought in the need for counselling. For those with substance abuse problems, participating in support groups that could reverse negative peer influences was a viable solution to psychological challenges. One of the female participants had tried various solutions like going to church and talking to friends but that did not put an end to her distress or problems.

*“I used to go to church and share with my girlfriends but I was not content. I also think peer counselling would also be good.”* 20-year old female.

*“I tried alcohol, cigarettes and generally going out for social events to feel ok but the pain was too deep in me. Especially after losing my dad and the insults I get from my brother. But the drinks did not help*….” 19-year old female participant.

Other participants who had been in psychotherapy before shared that it had a life-changing positive impact. Another client preferred psychotherapy as opposed to talking to friends and relatives. Some participants were concerned about the side-effects of medication and preferred psychotherapy as it presented no such risk.

*“I do not share my issues with other people. People are superficial and cannot be trusted. I prefer counselling. My friend had advised me to ask for anxiety drugs but I am not ready for medication…”* 22-year old male participant

We explored different strategies that participants had thought of and practiced to ease their pain. Those participants who had adjustment problems in school and got suspended on account of misconduct mentioned that they were more mindful of this and chose their friends carefully. Listening to parental advice, getting involved in extracurricular activities like sports, and making use of their talents were the strategies that the participants had put into consideration and practiced. They believed that this would not only make them better people, but also help them improve in school performance, time management, and forming bonds with people with whom they shared similar goals in life. Our participants alluded to the family therapy sessions that were organized to address interpersonal problems and so their challenges were relayed to their caregivers. One of our participant echoes this further:

*“If possible, I will ask my mum to come with me in next session. May be if the counsellor told her that I cannot be like her she will understand and stop being too harsh on me and having so high expectations form me.”* 9-year old male participant

#### Valuing psychopharmacological support in their overall treatment

While we found a lot of validation of the psychotherapeutic treatments our participants received, one male participant particularly emphasized his preference for medication as a form of treatment during the interview saying:

*“I am not a people person at all. Am hoping to be given some stress medicine and I will be good. Talking to people feels strange especially for a man. Men do not share their personal information.”* 20-year old male participant diagnosed with major depressive disorder.

## Discussion

We used a bifocal theoretical approach to guide this inquiry on attributions and private theories of mental illness amongst young adults. Weiner’s attributional model [16] guided our conceptual model as we looked at four domains: locus of control (internal vs. external), controllability of events (controllable vs. uncontrollable), stability of life circumstances (stable or unstable). Stigma (self/internal vs. external) was added as an independent domain given that mental illness can be highly stigmatizing in the Kenyan cultural context. The attributions were studied within the PTI-P [23]. In this process, we have tried to demonstrate that the attributional framework can help expand patients’ private theories/experiences about their problems and perceived solutions.

### Internal and external locus of control

In the present study, participants with an internal locus of control were relatively more resourceful in controlling their own behaviors once they were introduced to psychotherapy. The participants with an external locus of control do not have a determined role in shaping their response or energies towards a specific experience [36]. This implies that such individuals do not develop a sense of responsibility in establishing their own coping mechanisms and behavioral pathways, and hence their behaviors are shaped more in relation to the perceptions and interpretations of other people [37]. Consequently, we suspect, such individuals take longer to identify how the change could be made. Several studies have pointed to the interrelationship between increased levels of general self-efficacy, problem-oriented coping strategy, and internal locus of control as protective factors in bolstering mental health [38] and external locus of control is a good predictor of low mental health [39]. In a British study, one of the factors which facilitated the UK military personnel with post-traumatic stress disorder to engage in help-seeking behaviors was the sense of internal locus of control [40]. A case in point is that feelings of anger, fear, and thoughts of being unwell or the need to deal with one’s stressors are some of the internal/dispositional factors leading to treatment-seeking behavior. These participants were well in control of their feelings and thoughts. However, their psychological stressors had roots in some external, uncontrollable traumatic factors such as separation from parents, death of a loved one, and excessive stigmatization and discrimination from others. In Julian B. Rotter’s [41] explanation of external locus of control, events or outcomes depend on factors managed by environmental powers such as destiny or fortune outside of individual’s control [42]. The skills of problem-solving and positive thinking offered in therapy provided one mechanism to cope given these adverse circumstances in the lives of our participants. For instance, participants, who spoke of their childhood experiences or stigma from the public, viewed these challenges as stemming from an outward cause (external locus of control) rather than from within their own thoughts, feelings, or behaviors. Those who attributed psychological problems internally spoke more of their negative thoughts and feelings leading them to experience a psychological problem. It is likely that those with an external locus of control will experience greater challenges with problem-focused coping when stressed.

### Further implications of internal locus of control

Bitterness and hatred are internal processes. Each participant had a need for letting go of these emotional struggles [18]. Putting into consideration that these are within a person’s internal locus of control explains our *second conjecture that the internal locus of control might be positively associated with early positive engagement with one’s treatment such that it facilitates emotional regulation* and re-channeled our participants’ efforts in the face of external stressors. One of the participants, as quoted above, sought help on how to manage her anger. She shared that anger was the reason why she could not deal with daily life challenges but instead had outbursts that accelerated the problem.

### Stigmatizing contexts and relationships

Stigma can lead to excessive feelings of contempt and anger that triggers hostile behavior and other externalizing symptoms [43]. Unlike physical disabilities, persons with mental illness are perceived to be in control of their disabilities and be responsible for causing them. Furthermore, people are less likely to pity persons with psychiatric illness, instead reacting to psychiatric disability with anger and believing that help is not deserved [44]. This sentiment was also echoed by our research participants. For example, one of the participants attributed her psychological distress to being stigmatized by both family members and friends because of her epilepsy. Her father neglected her by not buying her medication and failing to pay her school fees. Another young participant shared that she had not told any of her friends or relatives about her decision to visit the clinic. She did not want people to know that she was seeking psychological help to avoid being labelled a mentally ill person.

Discrimination can also appear in public opinion about how to treat people with mental illness. For example, one client reported withdrawing from family functions due to stigma and discrimination from his immediate family members and relatives. It is worth noting that the behavioral impact (or discrimination) that results from public stigma may take four forms: withholding help, avoidance, coercive treatment, and segregated institutions [45]. Research also suggests that, instead of being diminished by the stigma, many persons become righteously angry because of the bias that they experienced [46]. This kind of reaction empowers people to change their roles in the mental health system, becoming more active participants in their treatment plan and often pushing for improvements in the quality of services [44]. It is due to these external attributions (stigma from others) that various participants we interviewed felt the need to seek therapy and were quite committed to it. Hence, it can be argued that the participants viewed this external attribution as an unstable attribution factor that could be changed through therapy. Thus this was a controllable attribution as well, since they thought that by being in therapy, they were at a higher position of controlling how they felt and even reacted towards stigma from others.

### Self-stigma and its links with attribution of controllability

An alternative reaction to anger about stigma is to turn prejudice inwards as self-discrimination. Research suggests self-stigma and fear of rejection by others lead people to quit pursuing life opportunities for themselves [47]. Self-esteem suffers, as does confidence in one’s future, as indicated by participants interviewed in this study. Some felt lost and wanted to find their place in society. An individual with mental illness may experience diminished self-esteem/self-efficacy, anger, or relative indifference depending on the parameters of the situation [48].

Cognitive theories of depression argue that beliefs of low self-worth and the tendency to attribute negative events to causes that are global (widespread rather than specific) and stable (will persist rather than change in the future) is associated with the development of depressed mood (Pearson et al. 2015). In our interviews, self-esteem was viewed as proportionally connected to the distress one experienced: the more the distress the poorer the self-esteem. One of our participants shared that she sought therapy to regain self-esteem and confidence. Therefore, self-stigma was an internal attribution that was viewed as a reason for seeking help since it was within the participants’ ability to be in control.

In the present study, prominent bio-psychosocial explanations of mental illness were identified from our ten participants. Previous research shows that patients tend to have more than one causal explanation for their mental illness [33]; an observation that echoes in our study too. Our participants attributed their problems to more than one cause.

Studies carried out in high-income countries [49] about public views regarding causes of mental illnesses reported that people predominantly held beliefs on mental illness to be social factors such as stressful life events, traumatic experiences, family problems, and social disadvantage [49–52]. Research carried out by Muga and Jenkins [3], Ikwuka et al. [53], and Samouilhan and Seabi [54] show that in Western contexts people might hold more biological explanations for their mental illness and increasingly seek medicines for these biological causes. However, in our study we noticed that whilst some of our participants had conditions that have biological determinants, the interface with therapy and work on psychosocial stressors remained the articulated needs of our participants.

Studies by Thwaites et al. [55] in UK and by Adewuya and Makanjuola, [56] in South Eastern Nigeria found that their participants attributed mental illnesses more to external than internal causes. Lingman and Lydén [33] found that causes such as poverty and negative family upbringing were common risk factors amongst young adults who sought psychological help. Environmental and social attributions have been identified as commonly seen stressors and our participants expressed similar concerns. In Ghana, for instance, participants mentioned issues such as unhealthy living conditions, lack of social support, relationships problems, society pressures, loneliness, and failure in life as reasons for becoming mentally ill [57].

A large community survey done in Nigeria [58] found that as many as one-third of the respondents suggested that possession by evil spirits could be a cause of mental illness, which was not the case in this present study. We suspect that as our work involved young adults’ under-30 years of age, the belief in spirit possession and traditional healing might not be as common as it might be in older people. More recent studies from Ethiopia showed inclusion of biological and psychosocial factors as explanations of mental disturbances in addition to the age-old spiritual and magical views [59]. Similarly a survey from a small town in Western Ethiopia reported psychosocial problems such as poverty, stress, and drug abuse as common explanations for mental illness in addition to explanations from religious/magical views such as God’s will or an attack by the evil spirit [60]. Another finding from North-western Ethiopia was that psychosocial and supernatural retribution were predominant explanations of mental illness, but less common for physical illnesses [61]. Mamah et al. [62] carried out a study on Kenyan youths’ perceptions about mental illness where they found that spiritual explanations were highly prevalent. However, in contrast to our study, the attribution to a spiritual cause was not alluded to, which is also similar to the findings by Ikwuka et al. [53]. This study was carried out at a public hospital based in the country capital and the participants interviewed had acquired a high school education with free access to the services offered at the hospital’s youth clinic; these factors may have influenced findings. In addition, we did not specifically explore spiritual attribution further in our interviewing.

Meyer and Garcia-Roberts [63] reported that the participants in their study preferred ‘sharing and talking through their distresses as a cathartic and helpful strategy.’ In our study, nine participants had tried other mediums of support such as prayers, focusing on their unique talent, avoiding bad company, and taking note of parental advice. In some ways, there was a development of an internal locus of control before they sought help from the health services. It could also be that there was a feeling that none of this could be sustained without adequate motivation and support that a professional could lend. This was a theme echoed by several participants of our study.

In a study from Pakistan, nearly half of the respondents reported psychiatric consultation to be the single most important management step [64]. This shows that people living in non-western countries endorse modern western medical care for mental health problems in addition to other more indigenous methods. Our participants tended to be contemplative and open-minded in seeking professional medical and psychosocial help for their mental illness, for which they were also willing to try various remedies for cure. Their views tended to be dynamic and agreeable to change, such as doing away with unwanted behavior that was a result of peer pressure in school. This is similar to other findings from non-western countries [65]. However, in our study it was evident that the private theories of several participants were influenced by Western views of pathogenesis and cure for mental illness. The apparent existence of Western conceptions could be the result of the participants being, what Sunday and Ibadan [66] describes as ‘transitional Africans’. Transitional Africans have received a Western education and, therefore, often incorporate both the African and Western values. We found this flexibility in thinking very heartening and felt that our participants understood their problems and appraised solutions in fairly multidimensional ways.

## Conclusions

Most research about etiological beliefs have investigated peoples’ beliefs about mental illness in general but there is virtually no scholarship on young peoples’ private theories of their own mental illness in Kenya. We have provided subjective explanations of Kenyan youths’ perceptions of their mental illness. Three key themes, psychosocial triggers of distress, biological conditions, and psychopathologies limiting interventions and subjective views on cure were private theories that we unpacked. When these private theories were mapped onto the attributional framework we imposed on the PTI-P we found that those who attributed their distress to an internal locus of control had a positive outlook towards therapy and behavior change. External factors were mainly ascribed to be the cause of negative emotions and thoughts leading to psychological illness. Stigma and self-stigma particularly were challenging attributions that needed socio-cultural awareness and youth empowerment work. Our limited data suggests that certain aspects of our participants’ lives emanated from uncontrollable events that shaped their locus of control to be externalized. Mental health care of young adults could benefit from exploration of their personal beliefs and attributions about their illness and cure in order to provide the best-adapted treatment for them and consequently make the mental health care more attuned to their concerns and needs.

### Limitations and next steps

We interviewed participants after they had a psychotherapy session with their therapist and diagnoses were already established by then. This might have influenced the clients’ thoughts and perceptions on mental illness and cure. However, the designed interview guide was structured in such a way that the client’s personal perceptions on these issues were explored, independent from therapist’s thoughts. The study was carried out in an urban setting with young adults, hence cannot be entirely generalized to youth living in rural or remote setting who may experience unique challenges in addition to their illness or those living in more marginal conditions, however the clinic serves as a referral for clients from all over the country. The experiences of our participants would most likely generalize to some other participants in the population.

We strongly feel that focusing on addressing experiences of young people in phenomenological ways offer insights into their psychosocial and intrapsychic processes. Future research efforts should be directed towards using this approach to understand attributions of mental illness in young people in diverse contexts, and more research is needed from resource-scarce context to understand mental health service implementation challenges. We believe that mental health needs of young people is an area requiring further phenomenological grounded theory based research. Such exercises would build an edifice of theoretical constructs useful for understanding what mental health, mental illness, and psychotherapies mean for young people in Kenya. And the findings from such studies may have wider resonance in other parts of Africa, too.

## Abbreviations

AFQ: Attributions Focused Question guide
ERC: Ethics Review Committee
HIV: human immunodeficiency virus
IPA: interpretative phenomenological analysis
KNH: Kenyatta National Hospital
LoC: locus of control
PTI-P: private theories interview-patient version
UK: United Kingdom
UoN: University of Nairobi
VCT: voluntary counselling and testing
YLD: years lived with disability

## Footnotes

1 (KCSE) Kenya Certificate of Secondary Education: End of high school level examination.
